# Experimental Analysis of Effect of Machined Material on Cutting Forces during Drilling

**DOI:** 10.3390/ma17112775

**Published:** 2024-06-06

**Authors:** Josef Sklenička, Jan Hnátík, Jaroslava Fulemová, Miroslav Gombár, Alena Vagaská, Aneta Jirásko

**Affiliations:** Department of Machining Technology, Faculty of Mechanical Engineering, University of West Bohemia, Univerzitní 22, 301 00 Pilsen, Czech Republic; sklenick@fst.zcu.cz (J.S.); jhnatik@fst.zcu.cz (J.H.); fulemova@fst.zcu.cz (J.F.); gombar@fst.zcu.cz (M.G.); anetam@fst.zcu.cz (A.J.)

**Keywords:** steel C45 (AISI 1045), case-hardened steel 16MnCr5, drilling, cutting forces, DOE, central composite design, predictive mathematical–statistical models

## Abstract

Current research studies devoted to cutting forces in drilling are oriented toward predictive model development, however, in the case of mechanistic models, the material effect on the drilling process itself is mostly not considered. This research study aims to experimentally analyze how the machined material affects the feed force (*Ff*) during drilling, alongside developing predictive mathematical–statistical models to understand the main effects and interactions of the considered technological and tool factors on Ff. By conducting experiments involving six factors (feed, cutting speed, drill diameter, point angle, lip relief angle, and helix angle) at five levels, the drilling process of stainless steel AISI1045 and case-hardened steel 16MnCr5 is executed to validate the numerical accuracy of the established prediction models (AdjR = 99.600% for C45 and AdjR = 97.912% for 16MnCr5). The statistical evaluation (ANOVA, RSM, and Lack of Fit) of the data proves that the drilled material affects the *F_f_* value at the level of 17.600% (*p* < 0.000). The effect of feed represents 44.867% in C45 and 34.087% in 16MnCr5; the cutting speed is significant when machining C45 steel only (9.109%). When machining 16MnCr5 compared to C45 steel, the influence of the point angle (lip relief angle) is lower by 49.198% (by 22.509%). The effect of the helix angle is 163.060% higher when machining 16MnCr5.

## 1. Introduction

The machining industry is constantly driven to increase productivity, optimize quality, and minimize the costs of the machining process [1,2,3,4]. To achieve these ends, it is necessary to have important knowledge of each machining technology, realize the research and development in the field of machine–tool–workpiece–fixture systems, and subsequently, implement the obtained knowledge into production [5,6]. Drilling is considered to be one of the most time-consuming operations, and it is estimated that up to 36% of all machine time is spent on holes’ production [7,8]. It is necessary to use high-throughput drilling tools to decrease the costs of drilling operations and significantly decrease the machining time [8], knowing that the magnitude of the tool force load and torque plays a key role when designing them [8,9,10]. Two approaches are used to determine the force load on the drilling tool: either direct measurement of the force load [11,12,13] or substitution into a mathematical model. Based on the classification provided by Song et al. in [14], models of cutting forces can be divided into four categories. Specifically: (i) macro-mechanical models based on the machining theory; (ii) mechanistic models which reflect the relationship between the cutting force, feed, cutting speed, tool geometry, and so on; (iii) micro-mechanical models describing the physical nature of machining; and (iv) numerical models developed by using the finite elements method (FEM) and discrete element method (DEM) to simulate the machining process. 

A macro-mechanical model was used by Priyanka et al. [15], where the formulas for calculation of feed force and torque were compiled (based on experiments with the drill diameter and cut depth as independent variables). The direct impact of the feed size on the feed force was demonstrated in [11,16,17]. In some cases, with an increasing cutting speed, the cutting force and torque increase, applying, e.g., deep hole drilling [18]. It is possible to influence the size of cutting forces by other parameters such as the process environment or the geometry of the tool. The team of Rodriguez et al. performed an experiment varying the point angle and cooling conditions [19]. Cutting force and torque increase with an increasing point angle of the drilling tool. 

Mechanistic models were designed by Parsian et al. [20] for torque, axis force (feed force), and cutting force in the plane perpendicular to the drilling axis. Anand et al. in [21], established a mechanistic model of cutting force effects during micro-drilling. A mechanistic model devoted to transient states was designed by Arif et al. [22] based on an analysis of the chip formation and local cutting forces along the main cutting edge. It is necessary to reach a relatively huge number of orthogonal tests to design equations for cutting forces and related specific cutting pressures, as Hamade et al. [23] reports. It is necessary to take into an account a wide range of cutting speeds, feeds, and rake angles. The studies [24,25,26,27,28,29] can be included in the field of micro-mechanical model application. FEM is an increasingly popular method to predict the impact of cutting conditions on machining performance [30]. This method has some issues nevertheless (e.g., the numerical implementation of major configuration changes) [31]. Material costs and maximizing productivity are the main benefits of FE analysis, and due to this, it is the target of many studies. Two-dimensional and three-dimensional FE models are designed to predict cutting forces [32,33,34,35,36]; the temperature of the tool and workpiece [30,32,33,34]; the shape of an emerging chip [37]; and residual stresses, etc.

A review of related works reveals weaknesses in terms of limitations in the application of the proposed models in many research and practice situations. Current studies of the cutting forces acting during drilling are mainly targeted toward predictive model development, but in the case of mechanistic models, mostly without taking into account the effect of the machined material on the drilling process. The microstructure of the machined material (carbon steels C05, C45, and C75) was studied in [37] while developing a 3D finite element computation model. The authors performed micro-drilling tests (with drills of different diameters) for the model verification regarding chip formation, torque, and feed force. Stainless steels are attractive materials due to their various applications, but high cutting forces and rapid tool wear occur during their machining/drilling. Therefore, many researchers are interested in the prediction and optimization of steel drilling parameters. For example, Ahmed et al. [38] used linear regression analysis to create empirical models predicting the responses (tool life, cutting force, and specific cutting energy) in relation to the defined control variables (the tool material, cutting speed, and feed rate) during the drilling of AISI 304 and AISI 2205. As stated in [39], the modeling of drilling (not only steel) is accompanied by various difficulties (changing conditions of the contact of the tool with the material) and requires considerable computing time, therefore, the development of techniques in mathematical modeling and simulation represents new research trends in this area [40]. Storchak et al. [39] dealt with the numerical modelling of drilling short holes in AISI 1045 material, and FEM and DOE were applied. Size effects during the micro drilling of carbon steel C45 are predicted by a 3D multiphase FE model in [41], and incremental hole drilling (AISI 1045 and CFRP) is modelled by FEM in [42]. In addition to these studies, the Deform 3D software is frequently used to simulate drilling operations in the literature [43,44,45], e.g., in [41], carbon steel AISI 1050 (DIN 1.1210) is the experimental material for the FE simulation of the drilling process, and drill stresses are analyzed by the Deform-3D. Artificial intelligence, multi-objective, and hybrid optimization of the drilling of AISI 1045 steel occur in the literature [46,47]. The examination of thrust force and torque remains a key issue in drilling processes [48]. Experiments devoted to the machining of the material 16MnCr5 (case-hardened steel) were carried out by Molnar [49] in order to achieve the selection of the most favorable cutting parameters based on the DOE, correlation analysis, and relative deviation analysis. The study [50] examined the fretting and plain fatigue behavior of case-hardened steel 16MnCr5, which is comparable to AISI 5115, as reported in [51]. 

Providing an adequate combination of experimental, numerical, and analytical approaches to modeling the drilling process is the object of this article. The main aim of the study is, therefore, to experimentally analyze the influence of machined material on changes in the value of the feed component of the cutting force *F_f_* (feed or thrust force) during drilling operation, with consideration of the influence mechanism of the chosen basic technological (*f_n_* and *v_c_*) and tool (*D*, *ε_r_*, *α_o_*, and *ω_r_*) factors on the monitored response (*F_f_*). We are interested in the statistically significant main effects and interactions of the chosen input variables. Based on the fact that machining processes are stochastic, the secondary aim of the study is to design a mathematical–statistical computational model providing a prediction of the influence of the technological and tool parameters on changes in the value of the feed component of the cutting force when drilling two different materials, C45 (EN 1.0503 and AISI 1045) and 16MnCr5 (C10E and EN 10132-2). The next aim is the subsequent implementation of experimental verification of the developed models. 

## 2. Theoretical Backgrounds

**Figure 1:**
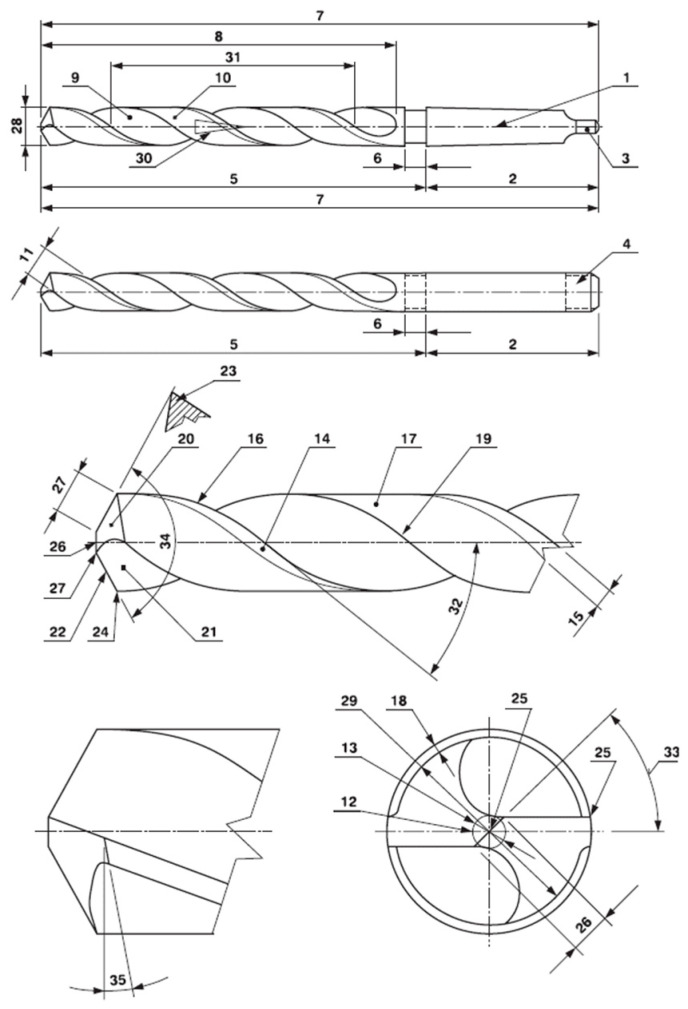
The standard nomenclature of a helical drill (1—axis, 2—shank or clamping part (conical, smooth cylindrical, or smooth cylindrical with a driver), 3—tang, 4—driver, 5—the drill body, 6—neck, 7—total length, 8—helix length of the groove, 9—groove, 10—body clearance surface, 11—the width of body clearance surface, 12—web (core), 13—web diameter, 14—margin, 15—margin width, 16—side cutting edge, 17—land relief, 18—land relief depth, 19—heel, 20—flank, 21—face, 22—main cutting edge, 23—wedge, 24—drill tip, 25—chisel, 26—chisel edge length, 27—main cutting edge length, 28—nominal tool diameter, 29—diameter of the relief, 30—reverse taper, 31—helix pitch, 32—helix angle, 33—chisel edge angle, 34—point angle, and 35—lip relief angle).

The authors of this study deal with the analysis of a drilling process with a helical (also called spiral or twist) drill, as shown in Figure 1. 

The geometric shape of the cutting part of the drilling tool influences the cutting process. The shape of the cutting edge (the lip) has an impact on the magnitude of the cutting forces, quality of the machined area, geometric accuracy, and tool wear [52,53,54]. The cutting force is decomposed for each cutting edge into three basic values influencing the tangential (cutting force *F_c_*), radial (passive force *F_p_*), and axial directions (feed force *F_f_*). The following relations are known: *F_f_* = *F_f_*_1_ + *F_f_*_2_; *F_p_ = F_p_*_1_ *+ F_p_*_2_; *F_c_ = F_c_*_1_ *+ F_c_*_2_; where *F_f_* is the feed component of the cutting force [N], *F_p_* is the passive component of the cutting force [N] and *F_c_* is the main component of the cutting force [N]. Their size depends on many factors such as the workpiece material, cutting depth, feed, and cutting tool geometry. The parameters affecting the cutting forces’ magnitude are the material characteristics of the workpiece; the parameters of the cutting process, i.e., the cutting conditions; and the geometric parameters of a helical drill. The magnitude of the cutting forces is influenced by the machined material, specifically, by its physical properties. The cutting process is evaluated according to the cutting environment (dry machining or when using cooling, etc.), further, according to the toughness of the whole assembly machine–tool–workpiece and to the cutting conditions (feed and cutting force). The tool angles represent geometric parameters [52,53]: side flank tool angle or lip clearance angle *α_o_*; tool tip angle or point angle *ε_r_*; main cutting edge tool angle *κ_r_*; helix pitch tool angle *ω_r_*; and side rake tool angle or chisel edge angle *λ_s_* [54,55,56]. The experimentally obtained Equation (1) is the most used formula for the cutting force *F_c_* determination [4]. In (1), the three most important factors are involved (in terms of the resulting effect), namely, the machined material, nominal diameter of the tool, and spindle feed rate.

(1) $$F_{c}=C_{F_{c}}D^{x_{F_{c}}}f_{ot}^{y_{F_{c}}},$$

where *C_Fc_* is a constant proportional to the influence of the machined material [MPa], *x_Fc_* is a constant appropriate to the drill diameter [–], *y_Fc_* is a constant expressing the influence of the spindle feed rate [–], *D* is the nominal diameter of the drilling tool [mm], and *f_ot_* is the feed per revolution [mm·rev^−1^]. Similarly, it is possible to calculate the feed force by Equation (2):

(2) $$F_{f}=C_{F_{f}}D^{x_{F_{f}}}f_{ot}^{y_{F_{f}}},$$

the meaning of *C_Ff_*, *x_Ff_*, *y_Ff_*, *D*, and *f_ot_* is analogously the same as mentioned above for Equation (2). The specific values of these constants are listed in Table 1.

## 3. Materials and Methods

### 3.1. Materials

Two types of material were used within the experimental part, i.e., unalloyed stainless steel for further refinement labelled as C45 (EN 1.0503 and AISI 1045) [57] and case hardening steel 16MnCr5 (1.7131, C10E, and EN 10 132-2) [58]. The C45 steel is suitable for the production of less-stressed machine parts in the refined state or after normalization annealing. The optimal mechanic values, including stiffness, are reached in a quenched and subsequently tempered state. C45 steel is suitable for surface quenching under blaze or induction, and oil quenching is preferred to prevent cracks for parts with a more complex shape. From the machining point of view (machinability), C45 is etalon steel with the machinability class 14b (or 13b). The specific chemical composition of the mechanic properties of the C45 steel is listed in Table 2.

**Figure 2:**
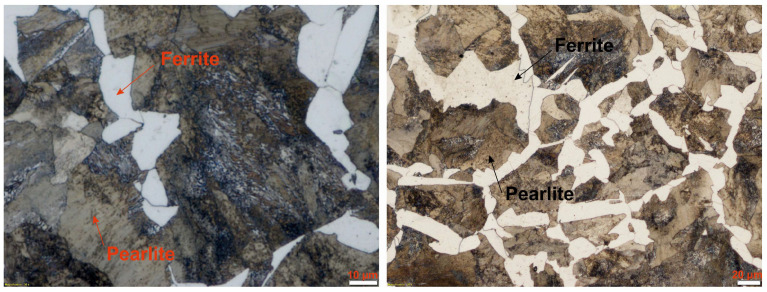
Microstructure of the used C45 steel samples.

The practical experimental verification of the chemical composition and mechanical properties of the used C45 samples confirmed the hardness values of *HV10*_C45_ = 221.500 ± 3.023 and the UTS of *R_m_*_,C45_ = 740.500 ± 1.447 MPa. As seen in Figure 2, the used material manifested a ferritic–pearlitic structure with the ferrite proportion of 17.489 ± 1.290%. 

A low alloy steel 16MnCr5 (also labelled as C10E, 1.7131, and EN 10 132-2) was the second experimental material, representing case-hardening steels [59] suitable for cementation. 16MnCr5 is widely used for moderately stressed machine parts and automobile components (gears and shafts, etc.) thanks to its mechanical properties [60]. The implementation of quenching and tempering appropriates its usage for components with a diameter of up to 35 mm max. It is suitable for welding and also for cold forming when suitably heat treated. Boron additive (0.0008 to 0.0050%) allows for increasing the toughness of the case-hardened layer. Within this study, the 16MnCr5 steel was used in the form of a round bar with a diameter of 120 mm, and its specific chemical composition and mechanical properties, determined experimentally, are listed in Table 3.

**Figure 3:**
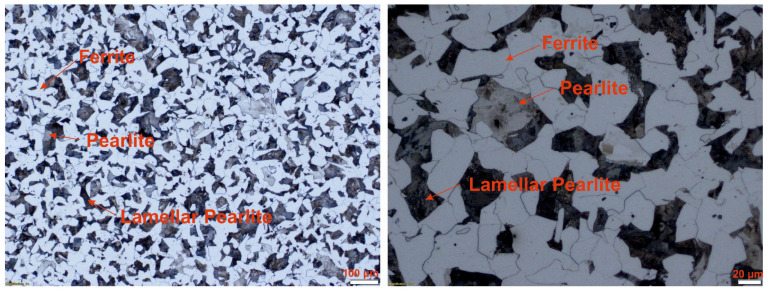
Microstructure of the used steel 16MnCr5.

The experimental verification of the chemical composition and mechanical properties of the used 16MnCr5 samples confirmed the hardness values of *HV10*_16MnCr5_ = 175.667 ± 2.267 and the ultimate tensile strength (UTS) of *R_m_*_,16MnCr5_ = 549.167 ± 10.894 MPa. As shown in Figure 3, the used material samples demonstrated a ferritic–pearlitic structure with ferrite proportion shares of 53.212 ± 1.975%.

### 3.2. Technological Parameters—Machine, Tool, Workpiece

**Figure 4:**
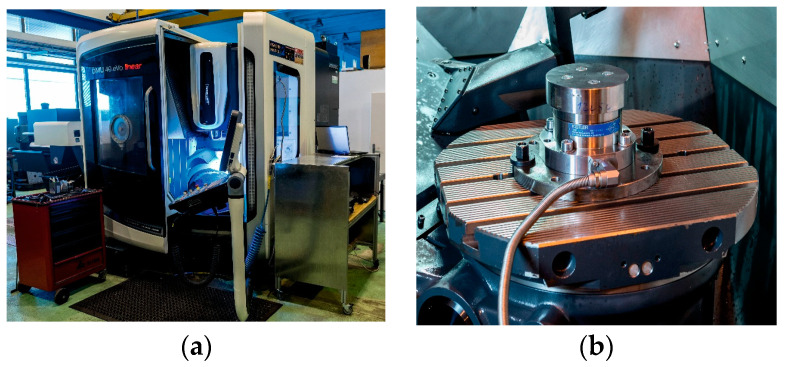
Photographs from experimental verification. (**a**) Machine and (**b**) workpiece clamped on the dynamometer.

The considered technological parameters were set as constant factors regarding the concept (principle) of the performed experiment. Namely: machine—DMU eVo40 linear (DMG, Bielefeld, Germany) (Figure 4a); workpiece (stock)—Ø 118 × 30 mm—ground; tool fixture—hydro plastic fixture with exchangeable housing for tools of diameters ranging from 8 to 12 mm; dynamometer (workpiece clamping)—dynamometer Kistler 9272 (Kistler, Winterthur, Switzerland), with the workpiece clamped to the landing surfaces of the dynamometer by screws with a conical head (Figure 4b). 

When setting the helical drill parameters as controlled factors, we focused on the macro geometry of the drilling tool. Micro geometric and surface parameters were set as constant factors. In the case of geometric factors, their independence is a critical condition, therefore, the input variables were divided into controlled (monitored) and constant factors (listed in Table 4). In terms of the aforementioned, the controlled factors were: *D*—nominal drill diameter [mm], ranging within the interval [8; 12]; *α_o_*—lip clearance angle in orthogonal plane [°], varying within the interval [8; 12]; *ω_r_*—helix angle in the basic plane [°], ranging within the interval [25; 35]; and *ε_r_*—point angle [°] within the interval [130; 145]. 

The drill tools were specially manufactured by the Regional Technological Institute (RTI), University of West Bohemia. The oil emulsion BLASOCUT BC 35 KOMBI from Blaser Swisslube CZ was used as the process liquid. The concentration was set to 6.9%, which corresponds to the manufacturer’s recommended range (from 6% to 8%). The used drilling tools were made from the CTS20D (Ceratizit, Garbsen, Germany) material coated by Triple Cr SHM with a slightly convex shape of the cutting edge.

### 3.3. Experimental Set Up

Taking into account that machining processes are multi-factor stochastic systems, where input factors affect changes in the investigated response not only as main effects, but also in interactions, the use of Design of Experiments (DOE) methodology is a natural and logical solution. This methodology, representative of a scientifically justifiable method of experimentation, provides a number of advantages [61]. For example, obtaining the maximum amount of information with the minimum number of performed experiments, the high numerical and statistical correctness of its obtained conclusions, and cost and time savings required for the implementation of experimental activities [61,62]. The statistical approach application is an appropriate solution when considering the stochastic nature of both the technological process (drilling) and the evaluated response (feed force).

A central composite design based on fractional replicates [62] was applied as a basic experiment design with the consideration of 6 input factors (4 tool factors and 2 technological factors). The resulting required number of measurements for one block was 46 (32 cube points, 2 central points, and 12 star points). When using central points, it is possible to verify whether the tests/measurements are performed correctly, i.e., whether there is no error during the actual measurement (e.g., due to the operation of the machine or measuring device). Each test (design point) is repeated six times to maximize the statistical value of the corresponding statement. This means that, for one combination of observed factors, we obtain six values for each dependent variable. The needed quantity of experimental tools follows from the compiled designed of the experiment. According to a combination of observed geometrical factors, 25 pieces are needed. In total, 2 pieces will be produced in each variant, so this means 50 pieces in total. There are 276 holes in total needed to drill for one bloc when using 25 different geometrically varying tools based on the above mentioned. It is also necessary to determine the appropriate number of stocks and the used pattern of the drilled holes to avoid an inappropriate influence of each measurement. The minimum wall thickness between each hole is set to 5 mm based on previous experience from experimental measurements of cutting forces during drilling operation. When targeting the size, 9 pieces of stock from each material are needed. One of them is prepared as a compensator when an operator error or measuring device failure occur. Table 5 reports the basic setting of the levels of individual factors. 

### 3.4. Processing of Experimentally Obtained Data

When performing individual experimental runs in accordance with the applied central composite design, the estimatation of the observed dependent variable (*F_f_* within this study) is generally described by a model in the form (3):

(3) $$\overset{⌢}{y}=b_{0}x_{0}+\sum_{j=1}^{N}b_{j}x_{j}+\sum_{\begin{array}{l} u,j=1\\ u\neq j \end{array}}^{N}b_{uj}x_{u}x_{j}+\sum_{\begin{array}{l} u,j=1\\ u\neq j \end{array}}^{N}b_{uj}x_{u}^{2}x_{j}+\sum_{\begin{array}{l} u,j=1\\ u\neq j \end{array}}^{N}b_{uj}x_{u}x_{j}^{2}+\sum_{j=1}^{N}b_{jj}x_{j}^{2}$$

where *b*_0_, *b_j_*, *b_uj_*, and *b_jj_* are appropriate regression coefficients and *x_j_* is an appropriate independent variable factor. Within our study, model (3) is used as the starting point of the second experimental step (DoE) and is aimed to predict the observed response *y* (*F_f_*) based on the changes in individual variables *x_i_* (the cutting speed, feed, cutting depth, and angle of the main cutting edge). The used general model (3) was subjected to statistical analysis: Analysis of Variance (ANOVA) and a Lack of Fit [61]. The ANOVA of the observed parameter *y* (*F_f_*) represents a statistical analysis of the suitability of the used general model (3). On the one hand, ANOVA enables analyzing whether the variability caused by random errors is significantly smaller than the variability of the measured values explained by the model. On the other hand, it allows for testing the null statistical hypothesis, which states that none of the effects used in the model (*D*, *f_n_*, *v_c_*, *ε_r_*, *α_o_*, and *ω_r_*) have a significant effect on changes in the investigated/measured variable *y* (*F_f_*). 

The Lack of Fit test is the second step during the analysis procedure of the suitability of the used general prediction model (3). For each *x_i_* factor setting (the level of each *i*-th factor), a group of experimentally obtained values is measured. Measurements within groups have some variability. The residuum variability is approximately similar to the variability within groups, if the regression model adequately describes (fits) the investigated dependence of *y* on *x*_i_. Thus, by comparing the variance of residuals and the variance within the groups, it can be determined whether the regression model adequately describes the dependence of *y* on *x_i_*. The excess variance of residuum in an improperly fitted model is called the Lack of Fit error. The experimentally obtained results (measured data) were subjected to the statistical analysis, methods, and procedures, due to the fact that a statistically designed experiment was applied in our performed experimental research. Moreover, the observed response (feed force *F_f_*) represents the measured value and can be understood as a random variable. It is burdened with gross, systematic, or random error, and the processing of the measured data in a non-statistical way would create the assumption of drawing erroneous conclusions and erroneous interpretations. 

## 4. Results and Discussion

### 4.1. Regression Model of Feed Force for Drilled Steel C45 and Results of Statistical Analysis

The basic statistical analysis of the used general model (3) to predict the value of *F_f_* (the observed response), depending on the changes in the values of the investigated (input and controlled) factors (*D*, *f_n_*, *v_c_*, *ε_r_*, *α_o_*, and *ω_r_*) for drilled steel C45, is presented in Table 6 (ANOVA) and Table 7 (Lack of Fit). As part of the analysis, there is a conclusion that this model explains 99.600% of the measured feed forces’ *F_f_* (response) variability based on the achieved value of the adjusted index of determination (*R_adj_*). The average value of the feed component of the cutting force *Ff* is 912.191 N, and the average error of the model is 18.711 N. 

The obtained results listed in Table 6 and the achieved significance level of *p* < 0.0001 enable us to proclaim that the variability caused by random errors is less than the variability of the values explained by the model, and, thus, at least one regression coefficient, significantly different from zero, exists within the model. So, regarding the Fisher–Snedecor test criterion and based on the aforementioned, the feed force prediction model for the C45 experimental material can be considered as adequate.

The Lack of Fit test (Table 7) is the second step when evaluating the regression model’s suitability. It is based on the zero statistical hypothesis stating that the residual variance is smaller or equal to the variance within groups. Based on the reached significance level of the model’s Lack of Fit test (*p* = 0.3935), it is possible to accept the zero hypothesis at the significance level of *α* = 0.05 and conclude that the used model sufficiently describes (fits) the monitored relationship.

After verifying the second component of the regression triplet, the model, the regression parameters of the model (3) can be correctly estimated (statistically and numerically). Table 8 lists only statistically significant regression coefficients (at the significance level of α = 0.05) which, in a significant manner, influence changes in the observed response *F_f_*. 

To accurately predict the response (the value of the feed component of the cutting force—*F_f_* for the drilled material C45) and capture interactions between the explanatory variables considered within this study, the experimentally obtained data were fitted to a multivariate non-linear regression model (MNRm) expressed by the response function $\overset{⌢}{y}=g(\overset{⌢}{x})$ based upon a response surface methodology. Our previous analyses led us to the assumed general model (3). Several variants of its form have been analyzed within the very selection of a specific form (4) of the regression model. When developing such a model (MNRm), the key task is to identify which subset of the *k* input variables is required to provide the best fit of the dependent variable. Table 8 provides an estimation of individual regression coefficients (in Estimate column), including the testing of their significance and VIF estimation. These coefficients are listed in the coded unit here (so unscaled), but they refer to the original measurement scale presented in Table 5. 

Based on Table 8, the absolute term of the regression model, i.e., Intercept (*b*_0_), is the most significant parameter of the regression model of the *F_f_* for the C45 material. Its influence on the change in the value of the observed response *F_f_* is at the level of 49.148%. This parameter *b*_0_ includes all effects that might have a significant influence on changes in the value of *F_f_*, but they were not considered as explanatory variables and were set as constant factors within the performed experiment (see Table 4). 

Let us evaluate the impact of the explanatory variables (according to Table 5) as the main effects, that is, without taking into account the Intercept (*b*_0_). Then, the most significant predictor is factor *x*_2_ (*f_n_*), with a 44.867% influence on the changing values of the feed force *F_f_*. Factor *x*_1_ (*D*) is the second significant predictor as the main effect with an influence share of 12.183% on *F_f_*. The next main effect demonstrates *x*_3_ (*v_c_*), with an influence share of 9.109% on the observed response. From the point of view of tool factors, *D*, *ε_r_*, *α_o_*, and *ω_r_* (according to Table 5), the most significant impact on changes in the *F_f_* value shows the factor *x*_4_ (*ε_r_*), with a share of 8.625%. The tool factor *x*_6_ (*ω_r_*) manifests an influence of 7.905%, followed by *x*_5_ (*α_o_*) with a 2.474% influence. 

Based on Table 8, it is obvious that mutual interactions of the observed factors also have a significant impact on changes in the *F_f_* value. The feed (*f_n_*), as the most significant main effect, also influences the *F_f_* in interaction with the nominal diameter of the drill *D* (3.432%), with the point angle *ε_r_* (3.650%), with the lip clearance angle *α_o_* (2.237%), and with the helix angle *ω_r_* (1.832%), and in mutual interaction with the point angle *ε_r_* and helix angle *ω_r_* (1.200%). The next significant interaction (*p* = 0.0076) with a significant impact on the feed force *F_f_*, is a combination of the point angle *ε_r_* with the clearance angle *α_o_* (1.325%), and also the squared value of the cutting speed *v_c_* (with an impact of 1.162% on the value changes of *F_f_*). 

In regard to the condition of the orthogonality of the experiment design, the analysis is performed with coded and dimensionless forms of the input variables (*x*_1_, *x*_2_, …, *x*_6_). To develop the regression model of the dependence of the feed force *F_f_* on the input factors (*D*, *f_n_*, *v_c_*, *ε_r_*, *α_o_*, and *ω_r_*) and express them in their natural scales, the DOE transformation was used [61]. Based on the estimation of regression coefficients, the application of DOE transformation, and subsequent modification, the prediction model of the feed component of the cutting force *F_f_* for the drilled material C45 can be written by Formula (4) as follows:

(4) $$\begin{array}{l} F_{f,C45}=74.869\varepsilon_{r}-238.594\alpha_{o}+93209.693f_{n}+387.336\omega_{r}+9.448v_{c}\\ +2.041\alpha_{o}\varepsilon_{r}-321.634\alpha_{o}f_{n}-620.678\varepsilon_{r}f_{n}-2.819\varepsilon_{r}\omega_{r}-2318.164f_{n}\omega_{r}\\ +493.0139f_{n}D-2.789\cdot 10^{-2}v_{c}^{2}-14.327D+16.107\varepsilon_{r}f_{n}\omega_{r}-11039.735 \end{array}$$

The analysis of residuals as the third component of the regression triplet is the last part of testing the correctness of the regression model MNRm (4). Based on the Shapiro–Wilks normality test (*p* = 0.447), the residuals show a Gaussian normal distribution with an average value of −1.740 × 10^−11^ N. Therefore, the developed mathematical–statistical model (4), describing the dependence of the observed parameter *F_f_* on the defined tool and technological factors (*D*, *f_n_*, *v_c_*, *ε_r_*, *α_o_*, and *ω_r_*), can be considered as the correct one, and, therefore, numerically and statistically appropriate. Regarding the aforementioned, it is possible to proclaim appropriate conclusions within the used intervals of the input factors. 

**Figure 5:**
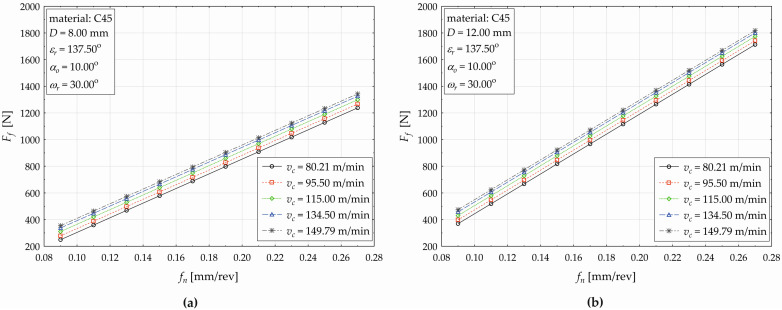
The impact of feed *f_n_* and cutting speed *v_c_* on the change in the value of the feed force *F_f_* (response) for the drilled material C45. (**a**) *D* = 8 mm and (**b**) *D* = 12 mm.

**Figure 6:**
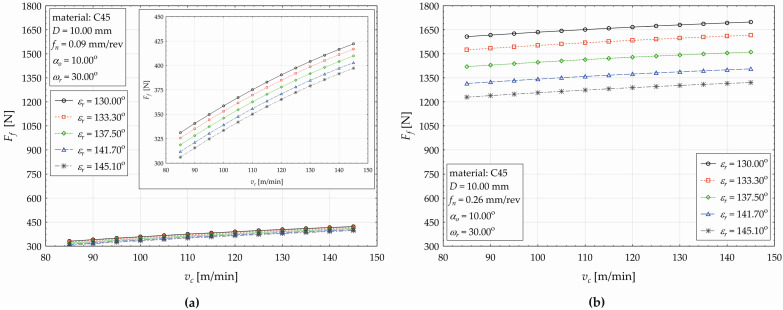
The effect of the cutting speed (*v_c_*) and the point angle (*ε_r_*) on the change in the value of response *F_f_* for the drilled material C45 when setting (**a**) *f_n_* = 0.09 mm·rev^−1^ and (**b**) *f_n_* = 0.26 mm·rev^−1^.

**Figure 7:**
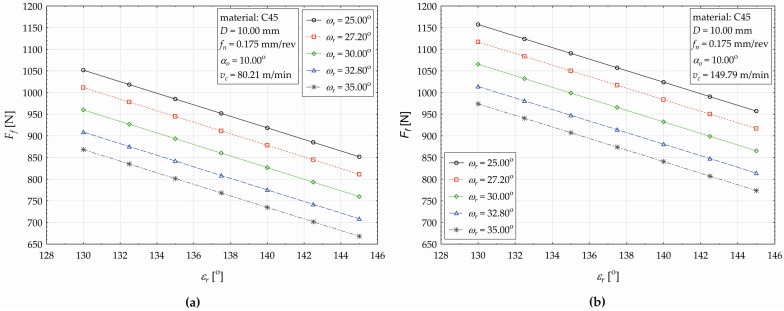
The effect of the point angle (*ε_r_*) and the helix angle (*ω_r_*) on the changes in the feed force (*F_f_*) values for the drilled material C45 when setting (**a**) *v_c_* = 80.21 m·min^−1^ and (**b**) *v_c_* = 149.79 m·min^−1^.

The developed regression model (4) allows us to plot the influences of the selected technological and tool factors on the response (*F_f_*), as shown subsequently in the next three figures (Figure 5, Figure 6 and Figure 7). Figure 5 demonstrates the feed (*f_n_*) impact on the feed force value (*F_f_*) when simultaneously varying the cutting speed (*v_c_*) for the used drill with diameters of *D* = 8 mm (Figure 5a) and *D* = 12 mm (Figure 5b) at constant values of *ε_r_* = 137.50°, *α_o_* = 10.00°, and *ω_r_* = 30.00°.

As shown in Figure 5, the value of response *F_f_* conditionally increases with an increasing feed (*f_n_*), and also increasing the cutting speed (*v_c_*) causes an increase in the values of *F_f_*, but the impact of *v_c_* is less pronounced (compared to the *f_n_* effect). When increasing the cutting speed from *v_c_* = 80.21 m·min^−1^ to *v_c_* = 95.50 m·min^−1^, the *F_f_* value increases by 28.98 N (it represents an increase by 11.628% for the drill with *D* = 8 mm in relative terms). When increasing the cutting speed (*v_c_*) from 95.50 m·min^−1^ to 115.00 m·min^−1^, the value of *F_f_* increases by 32.23 N (11.585%). When increasing *v_c_* from 115.00 m·min^−1^ to 134.50 m·min^−1^, the value of *F_f_* increases by 26.93 N (8.675%). When increasing the cutting speed from 134.50 m·min^−1^ to 149.79 m·min^−1^, the value of *F_f_* increases by 17.41 N (5.159%). Therefore, it can be generalized that, for a drill of *D* = 8 mm (*D* = 12 mm), an increase in cutting speed *v_c_* by 1 m·min^−1^ will cause an average increase in the feed force *F_f_* by 0.609% (0.411%), respectively. For a drill with a nominal diameter of *D* = 12 mm, the absolute value of the difference between the individual cutting speed levels is the same, but in relative terms, due to the higher value of *F_f_*, this difference is significantly lower. It fluctuates from 7.846% (when increasing *v_c_* from 95.50 m·min^−1^ to 115.00 m·min^−1^) to 3.804% (increase in *v_c_* from 134.50 m·min^−1^ to 149.79 m·min^−1^). A more significant difference in response (*F_f_*) values can be observed when simultaneously changing the drill diameter and increasing the feed. When setting the factor values at *v_c_* = 80.21 m·min^−1^ and *f_n_* = 0.09 mm·rev^−1^, the feed force value increases from 249.25 N (for a drill diameter of *D* = 8 mm) to 369.42 N (*D* = 12 mm). This increase by 120.18 N represents a relative difference of 48.217%. When setting the factor values at *v_c_* = 80.21 m·min^−1^ and *f_n_* = 0.11 mm·rev^−1^, the value of *F_f_* increases from 359.08 N (*D* = 8 mm) to 518.69 N (*D* = 12 mm), and the difference decreases to the level of 44.453%. When gradually increasing the feed *f_n_*, the difference value of *F_f_* between the used drill with *D* = 8 mm and *D* = 12 mm decreases (from 42.452% at *f_n_* = 0.13 mm·rev^−1^ to 38.389% at *f_n_* = 0.27 mm·rev^−1^). When setting the factor values at *v_c_* = 95.50 m·min^−1^ and *f_n_* = 0.09 mm·rev^−1^, the *F_f_* value increases from 278.23 N (*D* = 8 mm) to 398.41 N (*D* = 12 mm), which represents increase by 43.194% in relative terms. Next, increasing the cutting speed to 115.00 m·min^−1^ causes an increase in the feed force of 38.709%, increasing to 134.50 m·min^−1^ causes an increase of 35.619%, and increasing *v_c_* to 149.79 m·min^−1^ causes an increase in the *F_f_* value (for the drill with *D* = 12 mm) of 33.872%.

The effect of the cutting speed (*v_c_*) on the change in the feed force value (*F_f_*) when using five different levels of the point angle (*ε_r_*) is shown in Figure 6a for *f_n_* = 0.09 mm·rev^−1^ and in Figure 6b for *f_n_* = 0.26 mm·rev^−1^.

In general, based on the curves presented in Figure 6, it is possible to conclude for the drilled steel C45 that the response value (*F_f_*) conditionally increases with an increasing cutting speed (*v_c_*), and in addition, at higher values of the point angle *ε_r_*, the feed force values are higher. Of course, we can see differences in the course of the plotted functions for different feed settings, as at *f_n_* = 0.09 mm·rev^−1^ (Figure 6a), the non-linear change in the feed force *F_f_* is more pronounced than that at *f_n_* = 0.26 mm·rev^−1^ (Figure 6b). Let us evaluate the dependence of *F_f_* on the changing cutting speed *v_c_* for each setting of the point angle *ε_r_*. When setting *ε_r_* = 130.00°, the *F_f_* values are the highest and the relative increase in *F_f_* with an increasing cutting speed *v_c_* has a decreasing tendency. Specifically, during the drilling of C45 steel, if we increase *v_c_* by 5.00 m·min^−1^, starting from 85.00 m·min^−1^ and then *v_c_* ∈ {90, 95, …, 145}, the *F_f_* acquires corresponding values starting from 331.187 N and then *F_f_* ∈ {340.690, 349.844, …}, and the relative increase in the value of *F_f_* gradually reaches values from the set {2.869%, 2.687%, …, 1.361%}. Therefore, when the cutting speed *v_c_* is increasing, the conditional relative increase in the value of the feed component of the cutting force (*F_f_*) is not linear, it has a decreasing tendency. The average increase in *F_f_* represents 2.045%, and the application of model (4) makes it possible to conclude that, when increasing *v_c_* by 1.00 m·min^−1^, the value of *F_f_* increases by 0.034%. For *ε_r_* = 130.00°, the relative changes (increases) in the *F_f_* values with an increasing cutting speed *v_c_* fluctuate from 2.917% (*v_c_* = 90.00 m·min^−1^) to 1.379% (*v_c_* = 45.00 m·min^−1^). The average increase in the *F_f_* values represents 2.076%. As the value of the point angle *ε_r_* increases, the average increase in the feed force *F_f_* also increases, depending on the change in the cutting speed, namely by 2.116% (*ε_r_* = 137.50°), by 2.159% (*ε_r_* = 141.70°), and by 2.194% (*ε_r_* = 145.10°). Let us analyze the plotted functions in Figure 6a in the horizontal direction, i.e., for the chosen value of cutting force *v_c_*, let us analyze the changes in *F_f_* when changing the point angle in a basic plane. When setting *v_c_* = 85.00 m·min^−1^ and simultaneously varying the point angle *ε_r_* within the values set {130.00°, 133.30°, 137.50°, 141.70°, 145.10°}, the relative decrease in the value of *F_f_* gradually acquires corresponding values from the set {1.650%, 2.136%, 2.182%, 1.806%}. For example, it stands that an increase in the point angle from *ε_r_* = 133.30° to the level of *ε_r_* = 137.50° causes a relative decrease in the feed force *F_f_* by 2.136%. For example, at *v_c_* = 90.00 m·min^−1^, the relative decrease in the value of *F_f_* is by 1.604% (*ε_r_* = 133.30°) and 1.725% (*ε_r_* = 145.10°), while the maximum value decrease is by 2.119% for *ε_r_* = 141.70°. The average relative decrease in *F_f_* conditionally depends on the varying point angle *ε_r_* ranges from 1.944% (*v_c_* = 85.00 m·min^−1^) to 1.515% (*v_c_* = 145.00 m·min^−1^). 

The same principle was applied for the feed *f_n_* = 0.26 mm·rev^−1^ (Figure 6b), and the main difference (compared to Figure 6a) was in the percentage value of the relative changes. Based on the developed regression model (4) and calculated values of *F_f_*, the following can be stated: an increase in the cutting speed by 1 m·min^−1^ results in an average increase in the feed force within these values: by 0.094% (*ε_r_* = 130.00°) to 0.123% (*ε_r_* = 145.10°). When setting *f_n_* = 0.26 mm·rev^−1^ and *v_c_* = 85.00 m·min^−1^ and simultaneously varying the point angle *ε_r_* within the values set {130.00°, 133.30°, 137.50°, 141.70°, 145.10°}, the relative decrease in the value of *F_f_* gradually acquires corresponding values from the set {5.137%, 6.892%, 7.402%, 6.471%}. At *v_c_* = 90.00 m·min^−1^, the relative decrease in the *F_f_* value fluctuates, e.g., by 5.107% at *ε_r_* = 133.30°, by 6.425% (*ε_r_* = 145.10°), and its maximum is by 7.353% at *ε_r_* = 141.70°. For *f_n_* = 0.26 mm·rev^−1^, the average relative decrease in *F_f_* depending on varying the point angle *ε_r_* ranges from 6.475% (*v_c_* = 85.00 m·min^−1^) to 6.052% (*v_c_* = 145.00 m·min^−1^). The main difference between the used feed values *f_n_* = 0.09 mm·rev^−1^ (Figure 6a) and *f_n_* = 0.26 mm·rev^−1^ (Figure 6b) is in the absolute values of the feed component of the cutting force. The increase in the value of *F_f_* is more than 200% when using appropriate values of the cutting speed and point angle, so of course it fluctuates. For example, when changing the feed (from *f_n_* = 0.09 mm·rev^−1^ to *f_n_* = 0.26 mm·rev^−1^) at a constant *ε_r_* = 130.00° and *v_c_* = 85.00 m·min^−1^, there occurs an increase in *F_f_* by 385.420% (by 302.328% at *ε_r_* = 130.00° and *v_c_* = 145.00 m·min^−1^). The average difference in the feed force is 337.365% in the case mentioned above. When increasing point angle, this average difference of *F_f_* fluctuates from 321.658% (*ε_r_* = 133.30°) to 232.497% (*ε_r_* = 145.10°).

The effect of the point angle (*ε_r_*) on the change in the feed force value (*F_f_*) when using five different levels of the helix angle (ω_r_) is shown in Figure 7a for *v_c_* = 80.21 m·min^−1^ and in Figure 7b for *v_c_* = 149.79 m·min^−1^.

As seen in Figure 7, the response value (*F_f_*) conditionally decreases with an increasing the point angle value (*ε_r_*), and in addition, at higher values of the helix angle, the feed force values are lower. When setting the cutting speed *v_c_* = 80.21 m·min^−1^, helix angle *ω_r_* = 25°, and simultaneously increasing the point angle *ε_r_* in the values set {130.00°, 132.5°, 135°,145°}, the *F_f_* consequently decreases {1051.701 N, 1018.350 N, 984.999 N, 851.595 N}, and the relative decrease in the *F_f_* gradually acquires corresponding values {3.171%, 3.275%, etc.}. The total decrease in the feed force, when increasing the point angle from *ε_r_* = 130.00° to *ε_r_* = 145.00°, represents 19.027%. There is the conclusion that, at the listed values of the cutting speed and the angle, when the point angle increases by 1° (within the interval from *ε_r_* = 130.00° to the 145.0°), the conditional value of the feed component of the cutting force decreases by 1.268%. At the cutting speed of *v_c_* = 149.79 m·min^−1^ (Figure 7b), the same trends are observed. 

### 4.2. Regression Model of Feed Force for Drilled Steel 16MnCr5 and Results of Statistical Analysis

The statistical analysis of the used model (3) to predict the value of the feed force (*F_f_*), depending on varying the input technological and tool factors (*D*, *f_n_*, *v_c_*, *ε_r_*, *α_o_*, and *ω_r_*), is presented in Table 9 (ANOVA) and Table 10 (Lack of Fit). Based on the acquired value of the adjusted coefficient of the determination, this model explains 97.912% of the variability of the measured forces *F_f_*. The average value of the feed component of the cutting force (*F_f_*) for the material 16MnCr5 represents 1134.717 N, and the average error of the model reaches 55.186 N. 

Based on the results listed in Table 9 and the achieved level of significance *p* < 0.0001, it can be stated that the variability caused by random errors is smaller than the variability of the values explained by the model. This means that, in the model, there is at least one regression coefficient significantly different from zero. The predictive model for the feed force *F_f_* for the drilled material 16MnCr5 is, therefore, possible to consider as appropriate regarding the Fisher–Snedecor testing criterion. 

The achieved significance level (Table 10) of the Lack of Fit test (*p* = 0.4933) allows us to accept the zero hypothesis at the significance level of *α* = 0.05 and conclude that the used model sufficiently fits the monitored relationship. After verification of the model, the values of the statistically significant regression parameters of the model (3) for the drilled material 16MnCr5 can be correctly estimated, as listed in Table 11 (only significant factors and their interactions). 

As seen in Table 11, the most significant coefficient of the regression model predicting the feed force *F_f_* for the drilled material 16MnCr5 is Intercept (*b*_0_) with an influence of 45.558% (lower by 3.590% in comparison to the effect of *b*_0_ for C45). All effects that might have significant influence on the *F_f_* value might be included in *b*_0_, but within the performed experiment, they were set as constant factors (see Table 4). We evaluated the impact of the explanatory variables (Table 5) as the main effects, i.e., without consideration of the *Intercept* (*b*_0_). Based on the estimation of the regression coefficients, the application of DOE transformation [59], and subsequent modification, the prediction model of the feed component of the cutting force *F_f_* (influenced by *D—x*_1_, *f_n_—x*_2_, *v_c_*—*x*_3_, *ε_r_*—*x*_4_, *α_o_*—*x*_5_, and *ω_r_*—*x*_6_) for the drilled material 16MnCr5 was developed and can be written by Formula (5) as follows:

(5) $$\begin{array}{l} F_{f,16\mathrm{MnCr}5}=4434.989\alpha_{0}+164.675\varepsilon_{r}-168382.686f_{n}+3610.386\omega_{r}+7.840v_{c}-33.311\alpha_{0}\varepsilon_{r}-16658.399\alpha_{0}f_{n}\\ +1333.490\varepsilon_{r}f_{n}-363.152\alpha_{0}\omega_{r}-25.800\varepsilon_{r}\omega_{r}-44.800f_{n}v_{c}-7271.458\varepsilon_{r}f_{n}^{2}+839.532\alpha_{0}D+50.639\varepsilon_{r}D\\ +797.979f_{n}D+969869.535f_{n}^{2}-7325.586D-5.854\alpha_{0}\varepsilon_{r}D+121.152\alpha_{0}\varepsilon_{r}f_{n}+2.580\alpha_{0}\varepsilon_{r}\omega_{r}-22303.600 \end{array}$$

According to the outputs of the Shapiro–Wilks normality test (*p* = 0.930), the residuals show a Gaussian normal distribution with an average value of 4.980 × 10^−10^ N. Therefore, the developed mathematical–statistical model (5), describing the dependence of *F_f_* on the defined tools and technological factors (*D*—*x*_1_, *f_n_*—*x*_2_, *v_c_*—*x*_3_, *ε_r_*—*x*_4_, *α_o_*—*x*_5_, and *ω_r_*—*x*_6_), can be considered as the correct one (numerically and statistically appropriate), and it is possible to reach the correct conclusions within the used intervals of the input factors. Based on Table 11, the influence of the input factors as main effects was evaluated, and the percentage of their influence on the change in feed force was calculated. We present the results in order, starting with the most significant predictor (we indicate the decrease/increase in % compared to C45). Specifically, the order of the main effects of the input factors (Table 5) according to the size of their impact is: *x*_2_—*f_n_* 34.087% (decrease 10.780%) and *x*_1_—*D* 14.013%. The cutting speed (*x*_3_) has a significant impact on the response *F_f_* only in interaction with the feed (*x*_2_), and this interaction *x*_2_·*x*_3_ represents a 3.718% share of influence. The order of the main effects from the point of view of tools factors, *D*, *ε_r_*, *α_o_*, and *ω_r_* (Table 5), according to their effect size (in %) on the *F_f_* value when drilling samples of the 16MnCr5 material, is: *x*_1_ (*D)* 14.013%; *x*_5_ (*α_o_*) 8.839%; *x*_6_ (*ω_r_*) 6.125%; and *x*_4_ (*ε_r_*) 4.382%. Table 11 shows that the mutual interactions of the considered explanatory variables (*D*—*x*_1_, *f_n_*—*x*_2_, *v_c_*—*x*_3_, *ε_r_*—*x*_4_, *α_o_*—*x*_5_, and *ω_r_*—*x*_6_) also have a significant effect on the change in the value of the feed component of the cutting force *F_f_*. The influences of the interaction (in %) of the input factors are as follows: the squared value of feed *f_n_* occurs as interaction *x*_2_·*x*_2_ (4.566%) also with the point angle *x*_2_·*x*_2_·*x*_4_ (2.380%); feed (*x*_2_) interacts with the nominal diameter *x_2_*·*x*_1_(3.736%); *x*_2_·*x*_4_·*x*_5_—an interaction of the feed *f_n_*, point angle *ε_r_*, and lip clearance angle *α_o_* occurs (2.380%). The drill diameter (*D*) interacts with the lip clearance angle (*α_o_*) *x*_1_·*x*_5_ (3.958%); with the point angle (*ε_r_*) *x*_1_·*x*_4_ (3.450%); and there is interaction *x*_1_·*x*_4_·*x*_5_ (2.814%). The next interaction with a significant impact on the feed force *F_f_* that occurs is: the lip clearance angle (*α_o_*—*x_5_*) interacts with the point angle (*ε_r_*—*x*_4_) *x*_5_·*x*_4_ (2.952%); with the helix angle (*ω_r_—x*_6_) *x*_6_·*x*_5_ (2.445%), and together (*p* = 0.0002) *x*_4_·*x*_6_·*x*_5_ (3.155%) *p* = 0.0002. Comparing the drilled material 16MnCr5 to the C45, there are differences in terms of the character and effect sizes of the observed significant interactions (this will be mentioned in the Discussion section). 

**Figure 8:**
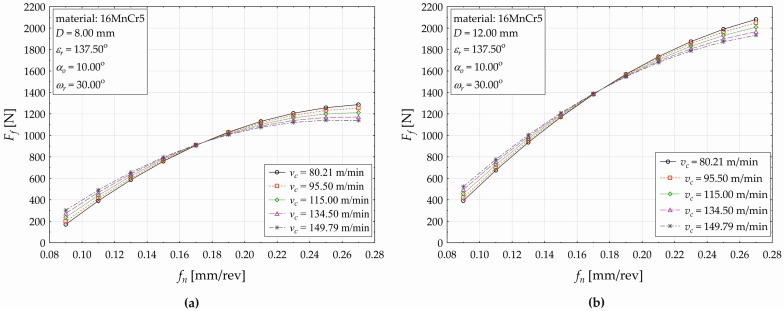
The impact of feed *f_n_* and cutting speed *v_c_* on the change in the value of the feed force *F_f_* for the drilled material 16MnCr5 using drill diameter of (**a**) *D* = 8 mm and (**b**) *D* = 12 mm.

**Figure 9:**
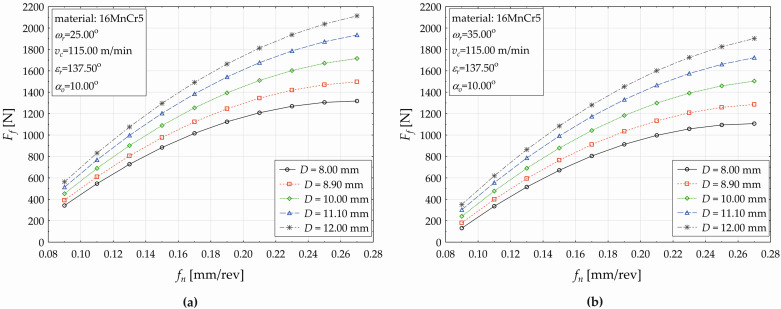
The effect of the feed *f_n_* and the drill diameter *D* on the changes in the feed force (*F_f_*) values for the drilled material 16MnCr5 when applying helix angle (**a**) *ω_r_* = 25.00° and (**b**) *ω_r_* = 35.00°.

**Figure 10:**
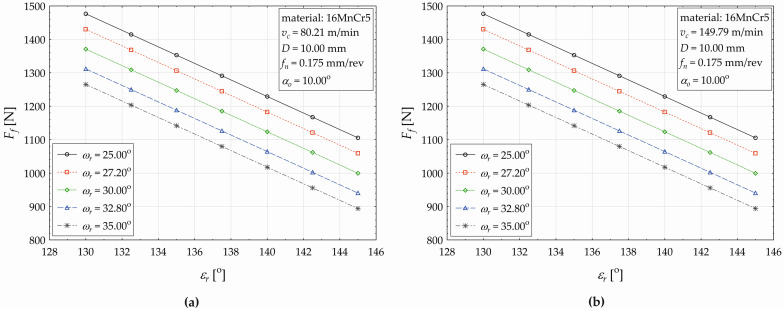
The impact of the point angle (*ε_r_*) and the helix angle (*ω_r_*) on the change in the response value of *F_f_* for the drilled material 16MnCr5. (**a**) *v_c_* = 80.21 m·min^−1^ and (**b**) *v_c_* = 149.79 m·min^−1^.

The developed regression model (5) allows us to plot the influences of the selected technological and tool factors on the response (*F_f_*) when drilling the 16MnCr5 material, as shown subsequently in the next three figures (Figure 8, Figure 9 and Figure 10). Figure 8 demonstrates the feed (*f_n_*) impact on the feed force value (*F_f_*) when simultaneously varying the cutting speed (*v_c_* —five levels) for the used drill diameters of *D* = 8 mm (Figure 5a) and *D* = 12 mm (Figure 5b) at constant values of *ε_r_* = 137.50°, *α_o_* = 10.00°, and *ω_r_* = 30.00°.

As shown in Figure 8, the value of the response *F_f_* conditionally increases with increasing the feed (*f_n_*). This increase in the value of *F_f_* has a significantly non-linear character (compared to the observed dependence when drilling the C45 at the same cutting conditions (Figure 6a). When setting the drill diameter *D* = 8 mm and constant cutting conditions as seen in Figure 8a, setting the cutting speed *v_c_* = 80.21 [m·min^−1^] and simultaneously increasing the feed *f_n_* [mm·rev^−1^] from the values set {0.09, 0.11, 0.13, … }, the *F_f_* consequently decreases {170.353N, 390.196N, 586.074N, …}, and the relative increase in the *F_f_* gradually acquires corresponding values {129.051%, 50.200%, …}. Further increasing the feed causes an increase in the *F_f_*, but the relative increase between the two adjacent applied feed values (Figure 8a) decreases (from 29.333% at *f_n_* = 0.13 mm·rev^−1^ to 2.236% at *f_n_* = 0.27 mm·rev^−1^). The nature of the value changes in the response when varying the feed is similar for the applied cutting speeds *v_c_* = 95.50 m·min^−1^ and *v_c_* = 134.50 m·min^−1^, but the gradual value change in *F_f_* is less significant. The change in the investigated functional dependence (*F_f_* on *f_n_*) is observed at the applied cutting force *v_c_* = 149.79 m·min^−1^. The effect of *f_n_* on the *F_f_* at each applied level of the cutting speed *v_c_* can be divided into two areas. When increasing the cutting speed within the defined range (Figure 8a), the conditional change in the *F_f_* value has a non-linear character, but increases within the interval (0.09, 0.17) of the feed *f_n_* [mm·rev^−1^] and decreases within the interval *f_n_* ∈ (0.19, 0.27). For example, increasing the cutting speed from *v_c_* = 80.21 m·min^−1^ to *v_c_* = 95.50 m·min^−1^, the value of *F_f_* decreases from 1286.205 N to the value of 1253.667 N (−2.530%). When applying the drill diameter *D* = 12 mm, the cutting conditions presented in Figure 8b, and varying the cutting speed *v_c_* at five defined levels, similar trends in the conditional response values are observed (non-linear character of the plotted curves), and there are differences in the absolute (and relative) value changes in the observed response (the feed force *F_f_*). The individual acquired values, as well as the percentage expressions of the increases/decreases in response values, are available on request from the authors.

The effect of the feed (*f_n_*) on the change in the feed force value (*F_f_*) when using five different levels of drill diameter (D) is shown in Figure 9a for the applied helix angle *ω_r_* = 25.00°and in Figure 9b for *ω_r_* = 35.00°.

As seen in Figure 9, it can be generally said that the response value (*F_f_*) conditionally increases with increasing the feed (*f_n_*) when using five different levels of drill diameter (D) and both levels of the helix tool angle *ω_r_* = 25.00° (Figure 9a) and *ω_r_* = 35.00° (Figure 9b). Moreover, when higher values of the drill diameter *D* are applied, lower feed force values are observed. The plotted functional dependences demonstrate the significant non-linear character in the value changes of the feed force (the response *F_f_*) for the observed mutual interaction of the two most significant main effects (*f_n_*, *D*) of the developed regression model (5). The observed values of *F_f_* are lower when higher values of the helix angle are applied, and this decrease fluctuates between −61.753% (*f_n_* = 0.09 mm·rev^−1^, *D* = 8.00 mm) and −16.039% (*f_n_* = 0.27 mm·rev^−1^, *D* = 8 00 mm). Increasing the nominal diameter of the drilling tool (*D*) causes a change in the decrease of the relative differences of the *F_f_* value between the used *ω_r_* levels (*ω_r_* = 35.00° and *ω_r_* = 25.00°), and it ranges between the value −37.527% (*f_n_* = 0.09 mm·rev^−1^, *D* = 12.00 mm) and the value −10.001 (*f_n_* = 0.27 mm·rev^−1^, *D* = 12.00 mm). For example, when increasing the drill diameter to *D* = 11.10 mm, the value of *F_f_* increases to 513.518 N, which represents an increase of about 13.421% (when compared to the applied diameter of *D* = 10.00 mm). More detail is presented in Table 12 and Table 13. 

To better explain the observed conditional change in the feed force (a relative decrease) depending on the two main effects (the point angle *ε_r_* and the helix angle *ω_r_*) when drilling the 16MnCr5 material, graphical representations of the observed relationship are displayed in Figure 10a (for *v_c_* = 80.21 m·min^−1^) and Figure 10b (for *v_c_* = 149.79 m·min^−1^).

In Figure 10, it is shown that, at both applied values of the cutting force *v_c_*, a decreasing trend of the response in *F_f_* is observed. For example, when setting the cutting conditions in terms of Figure 10b, the helix angle at the value of *ω_r_* = 25°, and increasing the point angle by 1° (within the considered interval from *ε_r_* = 130.00° to 145.00°), the total decrease in the feed component of the cutting force *F_f_* will represent 25.146%. This detailed analysis of the influence of the technological and tool factors (considered within the experiment) on the change in the value of the feed component of the cutting force (*F_f_*) is primarily aimed to compare the differences in their influences with respect to the machined material (C45, 16MnCr5). 

## 5. Discussion

**Figure 11:**
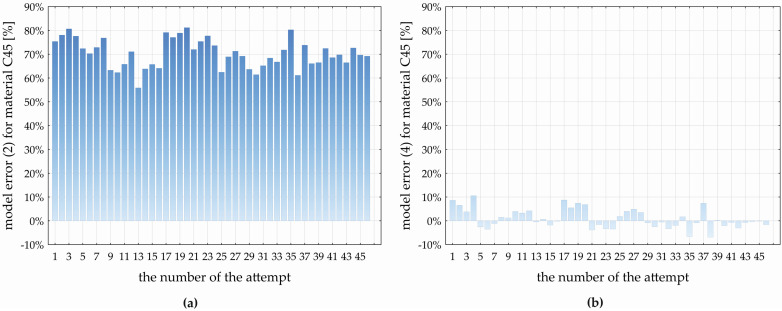
A relative error of the model predicting the feed component of the cutting force (*F_f_*) when drilling the material C45. (**a**) model (2) and (**b**) model (4).

**Figure 12:**
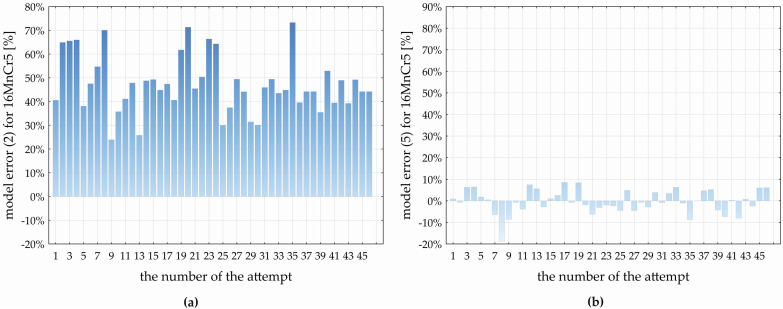
A relative error of the model predicting the feed force (*F_f_*) when drilling the material 16MnCr5. (**a**) model (2) and (**b**) developed model (5).

In the discussion section, we will provide an evaluation of the accuracy of the created prediction models (4) and (5) in comparison with Equation (2), applied in practice for the estimation of the feed component of the cutting force (*F_f_*) using the constants listed in Table 1, for both the investigated materials. We will primarily provide an analysis of the influence of the material itself, and its basic analyzed properties *Rm* and *HV10* on the change in the *F_f_* value based on a verification experiment. To more accurately predict the feed component of the cutting force (*F_f_*) during the drilling process of the materials C45 and 16MnCr5, multivariate non-linear regression models (4) and (5) were developed based on a statistical evaluation of the experimentally obtained data. Figure 11 and Figure 12 show the deviations between *F_f_* values obtained by the experiment and those calculated by models, i.e., deviations in the *F_f_* values predicted by models (2) and (4) for the C45 material (Figure 11a,b) and by models (2) and (5) for the 16MnCr5 material (Figure 12a,b). 

For the drilled material C45, model (2) shows an average relative deviation from the experimentally obtained *F_f_* values at the level of 70.365 ± 1.796% with a standard deviation of 6.048 ± 1.032% (Figure 11a). The minimum value of the relative error of model (2) represents 55.872% (*D* = 8.90 mm, *f_n_* = 0.22 mm·rev^−1^, *v_c_* = 134.50 m·min^−1^, *ε_r_* = 133.30°, *α_o_* = 8.90°, and *ω_r_* = 27.20°), and the maximum error of model (2) is 81.198% (*D* = 11.10 mm, *f_n_* = 0.13 mm·rev^−1^, *v_c_* = 95.50 m·min^−1^, *ε_r_* = 141.70°, *α_o_* = 11.10°, and *ω_r_* = 32.80°). The variation range of the error of model (2) represents 25.326%. Based on Figure 11a, the relative error of model (2) demonstrates: the lower quartile represents 65.787%, the upper quartile represents 75.372%, and the interquartile range is at the level of 9.858%. In comparison with the experimentally obtained *F_f_* values for C45, the developed MNRm (4) achieves an average relative error of 0.920 ± 1.229% with a standard deviation of 4.141 ± 0.706%. Based on Figure 11b, the maximum negative relative error of model (4) is −6.872% (*D* = 10.00 mm, *f_n_* = 0.18 mm·rev^−1^, *v_c_* = 149.79 m·min^−1^, *ε_r_* = 137.50°, *α_o_* = 10.00°, and *ω_r_* = 30.00°), and the maximum positive relative error represents 10.578% (*D* = 8.90 mm, *f_n_* = 0.13 mm·rev^−1^, *v_c_* = 95.50 m·min^−1^, *ε_r_* = 141.70°, *α_o_* = 11.10°, and *ω_r_* = 27.20°). The range of the relative error of model (4) represents 17.449%, the lower quartile is at the level of −1.931%, the upper quartile at 3.909%, and the interquartile range of the error of model (4) represents 5.840%. Based on the comparison of models (2) and (4) predicting the values of *F_f_* when drilling the C45 steel, it can be concluded that the experimentally developed model (4) shows an average error lower than model (2), namely, by 69.445%.

For the drilled material 16MnCr5, model (2) represents an average relative deviation from the experimentally obtained *F_f_* values at the level of 47.232 ± 3.556% with a standard deviation of 11.974 ± 9.932% (Figure 12a). The minimum relative error of (2) is 23.904% (*D* = 8.90 mm, *f_n_* = 0.22 mm·rev^−1^, *v_c_* = 95.50 m·min^−1^, *ε_r_* = 133.30°, *α_o_* = 8.90°, and *ω_r_* = 32.80°), and the maximum relative error is 73.279% (*D* = 10.00 mm, *f_n_* = 0.09 mm·rev^−1^, *v_c_* = 115.00 m·min^−1^, *ε_r_* = 137.50°, *α_o_* = 10.00°, and *ω_r_* = 30.00°). The relative error range of model (2) is 49.376%. Based on Figure 12a, the relative error of model (2) demonstrates that the lower quartile is 39.575%, the upper quartile is 50.364%, and the interquartile range reaches 10.789%. In comparison with the experimentally obtained *F_f_* values for the 16MnCr5, the developed MNRm (5) achieves an average relative error of 0.269 ± 1.903% with a standard deviation of 5.498 ± 0.938%. In Figure 12b, the maximum negative relative error of model (5) is—8.735% (*D* = 8.90 mm, *f_n_* = 0.13 mm·rev^−1^, *v_c_* = 134.50 m·min^−1^, *ε_r_* = 141.70°, *α_o_* = 11.10°, and *ω_r_* = 32.80°), and the maximum positive relative error of (5) is 8.578% (*D* = 11.10 mm, *f_n_* = 0.13 mm·rev^−1^, *v_c_* = 95.50 m·min^−1^, *ε_r_* = 133.30°, *α_o_* = 8.90°, and *ω_r_* = 32.80°). The range of the relative error of the developed model (5) represents 27.313%, the lower quartile is at the level of -3.079%, and the upper quartile of 4.709%. Based on the comparison of models (2) and (5) predicting the values of *F_f_* when drilling the 16MnCr5 steel, it can be concluded that the experimentally developed model (5) shows an average error lower than model (2), namely, by 46.963%.

A verification experiment was carried out to analyze the influence of the drilled material on the value change in the feed force (*F_f_*). The values according to Table 5 when setting constant factors in terms of Table 4 were used within each partial experimental test run. The partial experiments were not performed according to the DOE, but the influence of the selected technological (*f_n_*, *v_c_*) and tool (*D*) factors on the value change in the feed force (*F_f_*) was analyzed. The total number of tests reached 400, because each test was repeated *N*-times (*N* = 10) in the frame of the realized verification experiment. 

**Figure 13:**
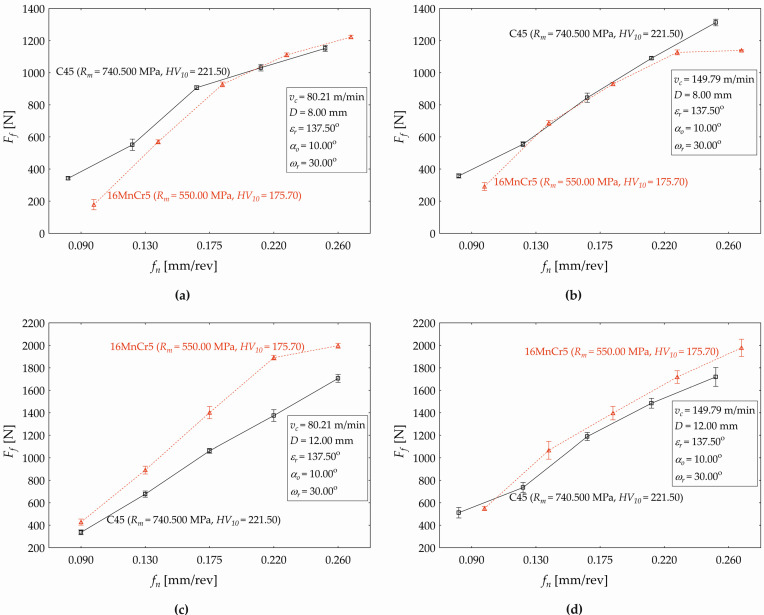
The influence of selected technological (*f_n_* and *v_c_*) and tool (*D*) factors on the change in the value of the feed force (*F_f_*) when setting (**a**) *v_c_* = 80.21 m·min^−1^, *D* = 8.00 mm; (**b**) *v_c_* = 149.79 m·min^−1^, *D* = 8.00 mm; (**c**) *v_c_* = 80.21 m·min^−1^, *D* = 12.00 mm; and (**d**) *v_c_* = 149.79 m·min^−1^, *D* = 12.00 mm.

Figure 13 presents the main changes in the feed force (*F_f_*) in the dependence on the feed (*f_n_*) that manifests the most significant effect on the response, in terms of the performed statistical analysis (Table 8 and Table 11) when drilling the both of materials (C45 and 16MnCr5 steels). The *f_n_* effect size is 44.867% (when drilling the C45) and 34.087% (when drilling the 16MnCr5) when applying the cutting conditions reported in Figure 13. Specifically, when setting a cutting speed of *v_c_* = 80.21 m·min^−1^ (Figure 13a,c), cutting speed at the value of *v_c_* = 149.79 m·min^−1^ (Figure 13b,d), and simultaneously setting the drill diameter at the level of *D* = 8.00 mm (Figure 13a,b) and the level of *D* = 12.00 mm (Figure 13c,d). Other tool factors such as *ε_r_* = 137.50°, *α_o_* = 10.00°, and *ω_r_* = 30.00° were constant during all the verification experiments. 

The performed analysis points out some interesting facts. The machined material has a significant impact on the value change in the feed component of the cutting force *F_f_* (*p* < 0.000). The average value of *F_f_* represents 796.660 ± 87.154 N when machining the material C45 and 801.551 ± 109.821 N when drilling the 16MnCr5 steel. This difference in the average values of *F_f_* is significant (*p* < 0.000) at the chosen level of significance *α* = 0.05 regarding the Scheffe test for both machined materials (C45 and 16MnCr5 steels). However, the material as a factor significantly (*p* < 0.000) impacts the change in the feed force value, even in interaction with the feed. When drilling the material C45, the feed force acquires the following average values (when setting the defined levels of the feed *f_n_*): 342.014 ± 2.246 N (*f_n_* = 0.09 mm·rev^−1^); 550.920 ± 8.439 N (*f_n_* = 0.13 mm·rev^−1^); 907.039 ± 2.939 N (*f_n_* = 0.175 mm·rev^−1^); 1031.390 ± 4.899 N (*f_n_* = 0.22 mm·rev^−1^); and finally, 1151.936 ± 4.359 N (*f_n_* = 0.26 mm·rev^−1^). When drilling the material 16MnCr5 (and setting the defined levels of the feed *f_n_*, the average value of the feed force acquires the following results. 177.894 ± 7.594 N (*f_n_* = 0.09 mm·rev^−1^); 569.312±3.017 N (*f_n_* = 0.13 mm·rev^−1^); 927.140 ± 3.581 N (*f_n_* = 0.175 mm·rev^−1^); 1111.028 ± 2.893 N (*f_n_* = 0.22 mm·rev^−1^); and 1222.381 ± 2.708 N at a feed of *f_n_* = 0.26 mm·rev^−1^. The differences in the average value of *F_f_* are significant (*p* < 0.000) at all the defined feed values *f_n_* [mm·rev^−1^], *f_n_* ∈ {0.09, 0.13s, 0.175, 0.22, 0.26} (Figure 13a). Therefore, the first conclusion can be stated, which is that the response of *F_f_* acquires higher values when machining C45 and setting the feed within the range *f_n_* ∈ {0.09, 0.13, 0.175}. However, when setting *f_n_* ∈ {0.22, 0.26}, the value of the feed force is significantly higher when machining the material 16MnCr5, specifically when applying the cutting speed *v_c_* = 80.21 m·min^−1^ and the nominal diameter *D* = 8.00 mm. 

Increasing the cutting speed to the level of *v_c_* = 149.79 m·min^−1^ (drill diameter *D* = 8.00 mm) resulted in the drilled material having no significant impact (*p* = 0.103) as the main effect on the change in the response (Figure 13b). The average value of *F_f_* is 831.775 ± 99.506 N when drilling the C45 steel and 834.590 ± 91.256 N when machining the 16MnCr5. The difference in the average values (0.341%) is not statistically significant (*p* = 0.989). The significant effect of the drilled material on the *F_f_* (*p* < 0.000) is observed in mutual interaction with the feed. The differences in *F_f_* values are significant (*p* < 0.000) at all defined feed values *f_n_* [mm·rev^−1^], *f_n_* ∈ {0.09, 0.13, 0.175, 0.22, 0.26} for the steel C45 and also for the 16MnCr5. When setting *v_c_* = 149.79 m·min^−1^ and *D* = 8.00 mm during drilling C45, the *F_f_* values are significantly higher, but only for *f_n_* ∈ {0.09, 0.26}. When changing the drill diameter and the cutting speed to the level of *D* = 12.00 mm and *v_c_* = 80.21 m·min^−1^ (Figure 13c), the machined material manifests the significant main effect (*p* < 0.000) on the feed force. In this case, the average value of *F_f_* is 1031.368 ± 139.602 N (for C45) and 1321.746 ± 170.864 N (for 16MnCr5). The difference in the average values of *F_f_* between both machined materials is also statistically significant (*p* < 0.000). As seen in Figure 13, the absolute average value of *F_f_* is higher when machining the material 16MnCr5 (by 29.049%) compared to C45 for all defined feed values. The maximum relative difference is observed at *f_n_* = 0.22 mm·rev^−1^ (37.511%) and the minimum at *f_n_* = 0.26 mm·rev^−1^ (17.079%). 

Figure 13d shows that, when applying the presented cutting condition and setting *v_c_* = 149.79 m·min^−1^ and *D* = 12.00 mm, the machined material (C45 and 16MnCr5) manifests a significant impact on *F_f_* as the main effect (*p* < 0.000), but also in mutual interaction (*p* < 0.000) with the feed. The average value of *F_f_* represents 1128.008 ± 129.383 N (C45) and 1342.024 ± 143.799 N (16MnCr5). The difference in the *F_f_* values between the machined materials (18.973%) is statistically significant (*p* < 0.000) at all defined feed values. Similar to the previous case (Figure 13c), the value of *F_f_* is statistically significantly higher when machining the material 16MnCr5 (compared to the C45) at all defined feed values *f_n_* ∈ {0.09, 0.13, 0.175, 0.22, 0.26}. The maximum relative difference is observed at *f_n_* = 0.13 mm·rev^−1^ (45.012%) and the minimum occurs at *f_n_* = 0.09 mm·rev^−1^ (7.424%). According to Figure 13a,d, it is necessary to notice that the machined material (C45, 16MnCr5) manifests a significant impact on *F_f_* also in a mutual interaction with the drill diameter (*D*) and the cutting speed (*v_c_*). When drilling the material samples and applying *D* = 8.00 mm and *v_c_* = 80.21 m·min^−1^, the average value of *F_f_* is 796.660 ± 87.154 N (for C45) and 801.551 ± 109.821 N (for 16MnCr5). The difference in *F_f_* average values between the machined materials is not statistically significant (*p* = 0.804) at these defined cutting conditions. When machining the material samples and setting *D* = 12.00 mm and *v_c_* = 80.21 m·min^−1^ (Figure 13c), the average value of *F_f_* is 1032 ± 139.040 N (for the drilled steel C45) and 1325.006 ± 170.870 N (16MnCr5). The difference in the average values of *F_f_* (28.287%) between both machined materials is statistically significant (*p* < 0.000) at the chosen significance level of *α* = 0.05. The tool factor (*D*) occurs as the key aspect in this mutual interaction influence (the drilled material, the drill diameter *D*, and the cutting speed *v_c_*). The differences in the average value of *F_f_* are not significant and could be considered as the stochastic influence when applying *D* = 8.00 mm, with no regard to the used cutting speed.

The performed verification experiment and analysis of the relationship *F_f_* = *y*(*f_n_*, *v_c_*, *D*, *ε_r_*, *α_o_*, and *ω_r_*), i.e., the impact of the considered technological and tool factors (Table 5) on the feed force *F_f_* when machining the C45 and 16MnCr5, outputs some interesting knowledge. The machined material is observed as the significant (*p* < 0.000) main effect (with a 17.600% share of impact) on the response (*F_f_*), moreover, its mutual interactions with the defined technological and tool factors are confirmed as statistically significant. Primarily, the impact of the feed (*f_n_*) on the *F_f_* represents 44.867% when machining the material C45, but a lower impact (34.087%) is recognized when machining the experimental material 16MnCr. The main differences occurred in the material effect on the *F_f_* when analyzing the cutting speed influence. The *v_c_* manifested a 9.109% share of significant impact on the *F_f_* when machining the material C45, but when machining the 16MnCr5, no significant effect of the cutting speed was demonstrated. The drill diameter demonstrated a 12.183% impact on the response (for the C45 material) and 14.103% when machining the material 16MnCr5. The influences of other considered tool factors (*ε_r_*, *α_o_*, and *ω_r_*) on the value change of *F_f_* demonstrate a significant dependence on the machined material. The impacts of the tool factors *ε_r_* and *α_o_* in the value change of the feed force are lower when machining the material with a lower strength value and lower hardness (16MnCr5) than when machining the material C45. Specifically, the point angle confirms (*ε_r_*) a 49.198% influence and the lip clearance angle in the orthogonal plane (*α_o_*) an influence of about 22.509%. On the other hand, the impact of the helix angle in the basic plane (*ω_r_*) on the change in the *F_f_* value is about 136.060% higher when machining 16MnCr5 compared to the steel C45. The material 16MnCr5 manifests a higher tensile strength and higher hardness. The mutual interaction of the point angle (*ε_r_*) and the helix angle (*ω_r_*) on the value change of *F_f_* is higher (about 122.759%) when machining the experimental samples of 16MnCr5 compared to the material C45. In addition to the above mentioned, different effects of mutual interactions of the tool and technological factors on the value changes in *F_f_* when machining the C45 (Table 8) and 16MnCr5 (Table 11) were observed. 

The achieved results are valid for the considered materials and defined cutting conditions, which limits the applicability of the developed mathematical–statistical prediction models to simulate the drilling of other materials. Modification of these models requires the implementation of additional experiments, which would correct the estimations of the regression coefficients of models for other drilled materials. The novelty of our research work lies in the complexity and scope of the considered explanatory variables. Above all, in that, unlike other publications [37,38,39,40,41,42,43], we also consider such tool factors (point angle—ε_r_, lip relief/clearance angle—α_o_, and helix angle—ω*_r_*) that meet the condition of mutual independence and the condition of orthogonality with respect to the DOE methodology. This article provides an adequate combination of experimental, numerical, and analytical approaches for hole drilling simulation. 

## 6. Conclusions

The authors of the research study provide an adequate combination of experimental, numerical, and analytical approaches to the modelling of the drilling process of two materials, namely C45 (*Rm* = 740.500 ± 1.447 MPa and *HV10* = 221.500 ± 3.023) and 16MnCr5 (*Rm* = 740.500 ± 1.447 MPa and *HV10* = 221.500 ± 3.023). The study is focused on the analysis of the machined material’s influence on the change in the value of the feed force *F_f_* during drilling operation, with consideration of the influence mechanism of the chosen basic technological (*f_n_* and *v_c_*) and tool factors (*D*, *ε_r_*, *α_o_*, and *ω_r_*). The defined main and parallel goals (to catch the statistically significant main effects and interactions of the chosen explanatory variables and develop mathematical–statistical regression models predicting the response) were achieved. For this purpose, two large experiments were carried out. The first one was carried out regarding the DOE methodology, and the obtained data were subjected to statistical analysis. The second experiment was performed to verify the obtained scientific results. Based on the aforementioned, the following conclusions can be proclaimed:

- an excellent agreement between the actual measured values of the investigated response (*F_f_*) and calculated *F_f_* values applying the developed regression model (4) for the machined material C45 (0.920 ± 1.229%) and (5) for the material 16MnCr5 (0.269 ± 1.903%) is demonstrated;
- the machined material is observed as the significant (*p* < 0.000) main effect (with a 17.600% share of impact) on the response (*F_f_*), moreover, its mutual interactions with the defined technological and tool factors are confirmed as statistically significant;
- the influence of the technological (*f_n_* and *v_c_*) and tool factors (*D*, *ε_r_*, *α_o_*, and *ω_r_*) on the response (the feed force) during the drilling process is necessary to monitor in mutual interactions with the machined material;
- the effect of the feed (*f_n_*) on the *F_f_* represents 44.867% when machining the material C45, but a lower impact (34.087%) is recognized when machining the experimental material 16MnCr;
- the *v_c_* manifests a 9.109% share of significant impact on the *F_f_* when machining the material C45, but when machining the 16MnCr5, no significant effect of cutting speed is demonstrated;
- the observed significant impact of the nominal diameter of the drilling tool on the response *F_f_* represents 12.183% when machining the material C45 and 14.103% when machining the material 16MnCr5;
- the influence of the point angle in the basic plane (*ε_r_*) on the value change of *F_f_* is about 49.198% lower when machining the material 16 MnCr5 compared to the material C45;
- the lip clearance angle in the orthogonal plane (*α_o_*) demonstrates about a 22.509% influence on the value change in *F_f_*;
- the impact of the helix angle in the basic plane (*ω_r_*) on the change in the *F_f_* value is about 136.060% higher when machining 16MnCr5 compared to the steel C45.

Despite certain conclusions that the effect of the drilled material on the change in the value of the feed component of the cutting force *F_f_* has not been proven [63], this research study confirmed that the material plays a significant role, both in terms of the size of *F_f_* and in terms of the mechanism of influence and mutual interactions of the technological (*f_n_* and *v_c_*) and tool (*D*, *ε_r_*, *α_o_*, and *ω_r_*) factors on the response (the feed force). The study itself has its limitations: it focuses on two types of steel (C45 and 16MnCr5), which may limit the generalization of the results achieved (to other materials with different mechanical and physical properties). The validity of the stated conclusions remains only for the proposed levels of the explanatory variables within the used intervals of the drilling cutting conditions. Limitations are also given by the used methodology and setting of the experiments, including the choice of controlled and constant factors. There is no such thing as a perfect experiment, so the effect of certain variables or conditions encountered in industrial applications (e.g., tool wear and machine vibration) is sometimes neglected/excluded in experimentation. This also presents limitations for the applicability of the results obtained. In our work, we focused on the regression models, but it would be interesting to apply modern methods of machine learning and tools of artificial intelligence. Taking into account the scope of the article, we did not use/present these methods in this case. For a comprehensive and more detailed analysis of the effect of individual factors on the change in the observed response (cutting force component), the authors of this study intend to use modern methods to process experimentally obtained data using machine learning methods (neural networks and decision trees, etc.). The authors already have experience with application of these methods, and they have implemented them in other research fields, e.g., [64]. 

Based on the experimentally obtained results, it could be expected that the material will have a significant impact on the value change in torque (*M_k_*) during drilling. Of course, observing the effect of the considered technological and tool factors on the value changes in *F_f_* and *M_k_* could give interesting results when machining other types of materials. The achieved results are valid for the defined materials and cutting conditions, and the modification of the models requires the implementation of additional experiments, which would correct the estimations of the regression coefficients of models for other drilled materials. The established mathematical–statistical models are applicable and will be used for prediction of the feed force in the CAM software (SolidCAM 2023 x64, SP3HF1) environment as a part of research projects at the University of Bohemia (CZ). 

## Figures and Tables

**Table 1:** Coefficient values used for cutting forces calculating.

Machined Material (Steel)	Constants					
	*C_Fc_*	*x_Fc_*	*y_Fc_*	*C_Ff_*	*x_Ff_*	*y_Ff_*
*R_m_* = 450 MPa	1570	1	0.78	440	1.10	0.55
*R_m_* = 600 MPa	1710	1	0.78	550	1.10	0.55
*R_m_* = 700 MPa	1840	1	0.78	630	1.10	0.55

**Table 2:** Chemical composition and mechanical properties of the drilled steel C45.

**Chemical Composition in Mass Percent**										
C%	Mn%	Si%	P%	S%	Cr%	Ni%	W%	Mo%	V%	Cu%
0.587	0.762	0.353	0.0117	0.0048	0.216	0.028	0.007	0.021	0.0032	0.0297
**Mechanical Properties of the Experimental Material**										
The test sequence number					1	2	3	4	5	6
Hardness *HV10* [-]					225	220	223	224	219	218
Ultimate Tensile Strength (UTS) *R_m_* [MPa]					741	742	741	738	741	740

**Table 3:** Chemical composition and mechanical properties of the drilled material 16MnCr5 (1.7131).

**Chemical Composition in Mass Percentages**										
C%	M %	Si%	P%	S%	Cr%	Ni%	W%	Mo%	V%	Cu%
0.196	1.06	0.203	0.0063	0.0238	0.945	0.0944	0.000	0.022	0.0017	0.1410
**Mechanical Properties of the Experimental Material**										
The test sequence number					1	2	3	4	5	6
Hardness *HV10* [-]					175	174	175	175	175	180
Ultimate Tensile Strength (UTS) *R_m_* [MPa]					561	532	548	547	548	559

**Table 4:** An overview of values of constant tool factors.

Constant Factors						
	Web Diameter	Margin Width	Supporting Bevel Width	Cutting Edge Radius	Drilling Depth	Chisel Edge Length
Unit	[mm]	[mm]	[mm]	[μm]	[mm]	[mm]
Value	0.3 *D*	0.115 *D*	0.135 *D*	15	2 *D*	0.025 *D*

**Table 5:** Setting of the levels of input factors according to the DOE.

Coded Factor	Factor	Unit	Factor Level				
			−1.784	−1	0	1	1.784
*x*_1_	*D*	[mm]	8.00	8.90	10.00	11.10	12.00
*x*_2_	*f_n_*	[mm·rev^−1^]	0.09	0.13	0.175	0.22	0.26
*x*_3_	*v_c_*	[m·min^−1^]	80.21	95.50	115.00	134.50	149.79
*x*_4_	*ε_r_*	[°]	130.00	133.30	137.50	141.70	145.00
*x*_5_	*α_o_*	[°]	8.00	8.90	10.00	11.10	12.00
*x*_6_	*ω_r_*	[°]	25.00	27.20	30.00	32.80	35.00

Note: *D*—nominal drill diameter, *f_n_*—revolution feed, *v_c_*—cutting speed, *ε_r_*—point angle, *α_o_*—lip clearance angle in orthogonal plane, and *ω_r_*—helix angle in basic plane.

**Table 6:** ANOVA of the prediction model of feed force *F_f_* for drilled material C45.

Source	df	Sum of Squares	Mean Square	*F* Ratio	*p*
Model	13	3,927,713.800	302,132.000	862.965	<0.0001 *
Error	32	11,203.500	350.000		
C. Total	45	3,938,917.300			

*—significant at the significance level of *α* = 5%. Source—source of the variance, df—number of degrees of freedom, Sum of Squares—sum of squared deviations, Mean Square—arithmetic mean of deviations, *F* ratio—calculated value of Fisher’s test statistic, and *p*—reached level of significance.

**Table 7:** Lack of Fit of the model of F_f_ for the material C45.

Source	df	Sum of Squares	Mean Square	*F* Ratio	*p*
Lack of Fit	31	11,105.489	358.242	3.6555	0.3953
Pure Error	1	98.000	98.000		
Total Error	32	11,203.489			

Note: Source—source of the error, df—number of degrees of freedom, Sum of Squares—sum of the squared deviations, Mean Square—arithmetic mean of deviations, *F* ratio—calculated value of Fisher’s test statistic, and *p*—the reached level of significance.

**Table 8:** Estimation of regression coefficients of the model of *F_f_* for the C45 steel.

Term	Estimate	Std Error	*t* Ratio	*p*	Lower 95%	Upper 95%	VIF
Intercept (*b*_0_)	920.852	4.430	207.85	<0.0001 *	911.828	929.877	.
*x*_2_	291.494	3.021	96.49	<0.0001 *	285.341	297.647	1
*x*_1_	79.146	3.021	26.20	<0.0001 *	72.993	85.299	1
*x*_3_	59.165	3.021	19.59	<0.0001 *	53.012	65.318	1
*x*_4_	−56.029	3.021	−18.55	<0.0001 *	−62.183	−49.877	1
*x*_6_	−51.359	3.021	−17.00	<0.0001 *	−57.513	−45.206	1
*x*_5_	−16.069	3.021	−5.32	<0.0001 *	−22.223	−9.917	1
*x*_2_·*x*_1_	24.404	3.308	7.38	<0.0001 *	17.667	31.142	1
*x*_3_·*x*_3_	−10.385	4.156	−2.50	0.0178 *	−18.851	−1.919	1
*x*_2_·*x*_4_	−25.980	3.308	−7.85	<0.0001 *	−32.718	−19.243	1
*x*_2_·*x*_6_	−13.031	3.308	−3.94	0.0004 *	−19.769	−6.294	1
*x*_2_·*x*_5_	−15.921	3.308	−4.81	<0.0001 *	−22.658	−9.183	1
*x*_4_·*x*_5_	9.429	3.308	2.85	0.0076 *	2.692	16.167	1
*x*_2_·*x*_4_·*x*_6_	8.524	3.308	2.58	0.0148 *	1.786	15.262	1

Note: *x*_1_—*D* [mm], *x*_2_—*f_n_* [mm·rev^−1^], *x*_3_—*v_c_* [m·min^−1^], *x*_4_—*ε_r_* [°], *x*_5_—*α_o_* [°], *x*_6_—*ω_r_* [°], *—significant at the significance level *α* = 0.05, Estimate—estimate of regression coefficient, Std Error—standard error of the estimation of a regression coefficient, *t* ratio—the calculated value of the Student’s test statistic, *p*—calculated value of the significance, Lower 95%—lower 95% reliability interval of the regression coefficient estimate, Upper 95%—upper 95% reliability interval of the regression coefficient estimate, and VIF—Variance Inflection Factors.

**Table 9:** Analysis of variance (ANOVA) for the prediction model of the feed component of the cutting force *F_f_* for material 16MnCr5.

Source	df	Sum of Squares	Mean Square	*F* Ratio	*p*
Model	16	6,475,432.500	404,715	132.887	<0.0001 *
Error	29	88,320.800	3046		
C. Total	45	6,563,753.300			

*—significant at the significance level of *α* = 5%. Source—source of the variance, df—number of degrees of freedom, Sum of Squares—sum of squared deviations, Mean Square—arithmetic mean of deviations, *F* ratio—calculated value of Fisher’s test statistic, and *p*—the reached level of significance.

**Table 10:** Lack of Fit of the developed model predicting *F_f_* for the drilled material 16MnCr5.

Source	df	Sum of Squares	Mean Square	*F* Ratio	*p*
Lack of Fit	28	86,916.338	3104.150	2.210	0.4933
Pure Error	1	1404.500	1404.500		
Total Error	29	88,320.838			

Note: Source—source of the error, df—number of degrees of freedom, Sum of Squares—sum of the squared deviations, Mean Square—arithmetic mean of deviations, *F* ratio—calculated value of Fisher’s test statistic, and *p*—the reached level of significance.

**Table 11:** Estimation of regression coefficients of the model predicting *F_f_* for the drilled material 16MnCr5.

Term	Estimate	Std Error	*t* Ratio	*p*	Lower 95%	Upper 95%	VIF
Intercept (*b*_0_)	1185.312	13.067	90.710	<0.0001 *	1158.587	1212.037	.
*x*_2_	329.193	8.909	36.950	<0.0001 *	310.970	347.415	1
*x*_4_	−103.969	21.871	−4.750	<0.0001 *	−148.701	−59.237	1
*x*_1_	135.374	8.909	15.190	<0.0001 *	117.152	153.596	1
*x*_6_	−59.182	8.909	−6.640	<0.0001 *	−77.404	−40.960	1
*x*_5_	−56.410	8.909	−6.330	<0.0001 *	−74.632	−38.188	1
*x*_2_·*x*_2_	−60.661	12.258	−4.950	<0.0001 *	−85.732	−35.589	1
*x*_2_·*x*_1_	39.500	9.756	4.050	0.0004 *	19.547	59.453	1
*x*_4_·*x*_1_	−36.500	9.756	−3.740	0.0008 *	−56.453	−16.547	1
*x*_4_·*x*_5_	31.188	9.756	3.200	0.0033 *	11.235	51.140	1
*x*_1_·*x*_5_	41.875	9.756	4.290	0.0002 *	21.922	61.828	1
*x*_6_·*x*_5_	−25.875	9.756	−2.650	0.0128 *	−45.828	−5.922	1
*x*_2_·*x*_3_	−39.313	9.756	−4.030	0.0004 *	−59.265	−19.359	1
*x*_2_·*x*_2_·*x*_4_	−61.844	23.949	−2.580	0.0151 *	−110.824	−12.864	1
*x*_2_·*x*_4_·*x*_5_	25.188	9.756	2.580	0.0151 *	5.235	45.140	1
*x*_4_·*x*_1_·*x*_5_	−29.750	9.756	−3.050	0.0049 *	−49.703	−9.797	1
*x*_4_·*x*_6_·*x*_5_	33.375	9.756	3.420	0.0019 *	13.422	53.328	1

Note: *x*_1_—*D* [mm], *x*_2_—*f_n_* [mm·rev^−1^], *x*_3_—*v_c_* [m·min^−1^], *x*_4_—*ε_r_* [°], *x*_5_—*α_o_* [°], *x*_6_—*ω_r_* [°], *—significant at the significance level *α* = 0.05, Estimate—estimate of regression coefficient, Std Error—standard error of the estimation of regression coefficient, *t* ratio—the calculated value of the Student’s test statistic, *p*—calculated value of the significance, Lower 95%—lower 95% reliability interval of the regression coefficient estimate, Upper 95%—upper 95% reliability interval of the regression coefficient estimate, and VIF—Variance Inflection Factors.

**Table 12:** Relative changes in the feed force (*F_f_*) value when increasing the drill diameter (*D*) at the applied helix angle *ω_r_* = 25.00° and setting the feed (*f_n_*) at specific values for the 16MnCr5 material.

*f_n_* [mm·rev^−1^]	*D* = 8.00 [mm]	*←D* = 8.90 [mm]	*←D* = 10.00 [mm]	*←D* = 11.10 [mm]	*←D* = 12.00 [mm]
0.09	-	14.525%	15.501%	13.421%	9.681%
0.11	-	11.725%	12.826%	11.368%	8.352%
0.13	-	10.792%	11.906%	10.639%	7.868%
0.15	-	10.509%	11.622%	10.412%	7.716%
0.17	-	10.553%	11.667%	10.448%	7.740%
0.19	-	10.813%	11.927%	10.656%	7.879%
0.21	-	11.247%	12.356%	10.997%	8.106%
0.23	-	11.843%	12.942%	11.459%	8.411%
0.25	-	12.612%	13.688%	12.040%	8.792%
0.27	-	13.582%	14.615%	12.751%	9.253%

**Table 13:** Relative changes in the feed force (*F_f_*) value when increasing the drill diameter (*D*) at the applied helix angle *ω_r_* = 35.00° and setting the feed (*f_n_*) at specific values for the 16MnCr5 material.

*f_n_* [mm·rev^−1^]	*D* = 8.00 [mm]	*←D* = 8.90 [mm]	*←D* = 10.00 [mm]	*←D* = 11.10 [mm]	*←D* = 12.00 [mm]
0.09	-	37.976%	33.640%	25.172%	16.454%
0.11	-	19.118%	19.617%	16.400%	11.527%
0.13	-	15.218%	16.143%	13.899%	9.984%
0.15	-	13.815%	14.835%	12.919%	9.361%
0.17	-	13.327%	14.373%	12.567%	9.134%
0.19	-	13.318%	14.365%	12.560%	9.130%
0.21	-	13.631%	14.661%	12.787%	9.276%
0.23	-	14.210%	15.207%	13.199%	9.540%
0.25	-	15.049%	15.987%	13.783%	9.911%
0.27	-	16.176%	17.018%	14.543%	10.388%

## Data Availability

Data are available based upon the request.
